# Evaluation of Garlic Landraces from Foggia Province (Puglia Region; Italy)

**DOI:** 10.3390/foods9070850

**Published:** 2020-06-29

**Authors:** Anna Bonasia, Giulia Conversa, Corrado Lazzizera, Pasqua Loizzo, Giuseppe Gambacorta, Antonio Elia

**Affiliations:** 1 Department of the Science of Agriculture, Food and Environment (SAFE), University of Foggia, 71100 Foggia, Italy; anna.bonasia@unifg.it (A.B.); corrado.lazzizera@unifg.it (C.L.); antonio.elia@unifg.it (A.E.); 2 Department of Soil, Plant and Food Sciences, University of Bari, 70126 Bari, Italy; pasqualoizzo@gmail.com (P.L.); giuseppe.gambacorta@uniba.it (G.G.)

**Keywords:** *Allium sativum* L., agro-biodiversity, local varieties, bulb morphology, phenols, volatile compounds

## Abstract

Interest in local landraces has unfortunately decreased over, the last decades, in which they have been continuously subjected to a high genetic erosion in favour of new modern varieties. Within the Puglia region (S-E Italy), Foggia province was found to be the richest in vegetable landraces. In the present study, six garlic landraces collected from this area have been assessed for their chemical composition (minerals, organic acids, free sugars, volatile, and phenolic compounds) along with their main morpho-biometrical traits. A commercial genotype was also considered as a reference standard. The landraces show a large variability, but in general high morphological standards, high levels of cations and phenols, and low levels of volatile-(S)-compounds in comparison with the commercial genotype and the literature values. ‘Aglio di Peschici’ and ‘Aglio Rosso di Monteleone di Puglia’ are very rich in minerals and phenols (mainly ferulic acid and iso-rhamnetin). This increase in knowledge on the chemical properties of these garlic landraces could represent a tool for encouraging the consumption of a food product. At the same time, the consumption of these landraces would stimulate their cultivation and could highly contribute to protection against the risk of erosion of agro-biodiversity by their in situ/on-farm conservation.

## 1. Introduction

Garlic (*Allium sativum* L.) is one of the most important bulb vegetables and is mainly used as a spice or flavouring agent for foods. It is used in several types of products such as garlic oil, powder, salt, paste, and flakes.

Garlic has a high nutritive value as a rich source of minerals, carbohydrates, proteins, and vitamins. It also contains numerous bioactive molecules such as organic sulphur-containing (S-compounds) compounds and phenolic compounds [1,2,3].

The organic S-compounds (present in intact garlic cloves are γ-glutamyl cysteine and cysteine sulfoxides, including deoxyallin and mainly alliin [4]. When garlic is mechanically damaged, the vacuolar enzymes (allinase) convert alliin to allicin. Unstable allicin undergoes non-enzymatic degradation to form a variety of volatile S-compounds—ajoene, vinyl-dithiins, and sulphides as mono-, di-, and tri-sulphides [4]. Numerous studies have reported that the organic S-compounds are responsible for the biological activities revealed in different pathological situations [1,2,5].

Garlic is also characterized by phenolic compounds and the main group consists of phenolic acids (mainly as caffeic acids), followed by flavonoids (mainly as quercetin) [6,7,8]. A lot of research has been conducted to assess the dietary role of phenolic compounds, their characteristics, metabolic pathways, and biological effects, affirming their capacity to scavenge Reactive Oxygen Species (ROS) and to treat a variety of diseases including heart disease and cancer [2,6].

The Puglia region (South-East Italy) in the centre of the Mediterranean basin has a rich agro-biodiversity, with particular reference to landraces (also known as ‘local varieties’ and ‘farmer’s varieties’). Landraces are variable, identifiable populations, usually known by a local name, lacking ‘formal’ crop improvement, and characterized by a marked adaptation to specific environmental conditions of the area of cultivation [9].

Unfortunately, the regional agro-biodiversity of vegetable crops has been partially lost due to several factors. The modernization and intensification of agriculture have resulted in vast agro-biodiversity loss, since farmers grow modern varieties and hybrids and tend to abandon traditional landraces. 

As a result of a regional Project (https://biodiversitapuglia.it//), aimed at collecting and identifying vegetable resources at risk of genetic erosion, the province of Foggia was found to be the richest in landraces among the five provinces of the region [10]. In the *Alliaceae* botanical family the most frequent species found was garlic (*Allium sativum* L.). To stimulate the cultivation of landraces from the farmers (‘keepers’), their consumption has to be promoted. To do this, the identification and the highlighting of the chemical properties of the landraces compared with the ‘commercial’ varieties may represent an added value of the product that could push the preference of consumers. 

In this view, the present study describes for the first time the chemical properties of six landraces collected in the province of Foggia, along with their main morpho-biometric traits.

## 2. Materials and Methods 

### 2.1. Description of Collecting Sites, Plant Materials, and Sampling

Six landraces of garlic (*Allium sativum* L.) were directly collected from local growers which had very small farms in marginal areas, in the province of Foggia, in the North of Puglia region (Italy), which lies geographically between 41°54′44′′N and 41°02′27′′N in latitude and 14°55′58′′E and 14°02′27′′E in longitude. All the interested growers had very small farms in marginal areas and all of them adopted low input agronomic practices such as minimum soil tillage, hand weed control and scarce or no use of irrigation, pesticides or fertilizers.

Details of the collected landraces and the collecting sites are presented in Table 1. Bulbs of ‘Spanish white garlic’ (‘Palmieri Import’, Afragola (Na); Origin: Spain), widely available on the European and national market, was used as a reference standard. Pictures of the examined genotypes are shown in Figure S1.

Samples of bulbs (3 ± 0.5 kg per each genotype) were well mixed to obtain three independent replicates, each consisting of 15–40 bulbs. Morphological and biometrical measurements on cloves and bulbs were carried out on fresh material, dealing with 7–15 bulbs for each replicate. Chemical analyses of peeled cloves were performed in triplicate for each replicate, after being freeze-dried (CoolSafe SCANVAC, Labo0Gene, Allerød, Denmark), powdered, packed in hermetic jars and then stored in the dark at −18 ± 1 °C until the analyses were carried out. 

### 2.2. Appearance and Morpho-Biometrical Measurements

The description of the morphological features for each genotype was done following the descriptors 7.1.11 (bulb shape), 7.1.20 (bulb structure type), 7.1.16.1 (bulb outer skin colour), and 7.1.16.2 (skin colour of the clove), provided by the International Plant Genetic Resources Institute (IPGRI) guidelines [11]. The main biometrical traits for each genotype were assessed. A dry matter concentration of the peeled cloves was calculated after freeze-drying the material.

Measurements of colour on external bulbs (with dry tunics) and cloves (with thin skin) were also performed by images analysis using an image acquisition station (Immagini & Computer, Bareggio, Milano, Italy), equipped with four white lamps, camera NIKON D5200, and software Image Pro Plus 7.0 (Media Cybernetics, Inc., Rockville, MD, USA). Colour indices were based on the CIELAB scale 1976: L*, indicating lightness/darkness, ranging from 0 (black) to 100 (white) value in a grey scale; a* reflecting greenish (if negative) to reddish (if positive) tonality; b* indicating bluish (if negative) to yellowish (if positive) tonality. In addition, the derived parameters, hue angle (h°), and chroma (C*), indicating respectively the hue and the vividness/dullness, were evaluated.

### 2.3. Chemical Measurements

#### 2.3.1. Standards and Reagents

HPLC-grade methanol, metaphosphoric, formic, methanesulfonic acid, acetonitrile, ethanol were purchased from Sigma-Aldrich (St. Louis, MO, USA). NaOH, sodium carbonate, and sodium bicarbonate were purchased from Merck KGaA (Darmstadt, Germany). 

Standards for phenolic (caffeic acid, caftaric acid, quercetin 3-ß-d-glucoside, quercetin, rutin, hyperoside) and volatile (di-allyl-sulphide-DAS; di-allyl-disulphide-DADS; di-allyl-trisulphide-DATS, di-methyl-sulphide, crotonaldehyde, pelargonaldehyde) compounds were purchased from Sigma-Aldrich (MO, USA). 

Glucose, fructose, and sucrose standards were purchased from Chem Service (West Chester, USA); all the other standards were purchased from Sigma-Aldrich (MO, USA). Ultrapure water (18.2 MΩ cm resistivity at 25 °C) was obtained from a water purification system Milli Q (Millipore, Germany).

#### 2.3.2. Minerals

Ashes were determined by a muffle furnace according to the AOAC method 923.03. 

Inorganic ions were analyzed by ion chromatography (Dionex ICS 3000; Dionex-ThermoFisher Scientific, Waltham, MA, USA). Inorganic cations were extracted from lyophilised samples (1 g), previously ashed (in a muffle furnace at 550 °C for 6 h) and acid digested (20 mL of 1 mol L^−1^ HCl in boiling water for 30 min), before injection into the ion chromatography system. For inorganic anions, the lyophilized samples (0.5 g) were extracted with 50 mL of an eluent solution (3.5 mM sodium-carbonate and 1.0 mM sodium-bicarbonate) in a shaking water bath at room temperature for 30 min. The mixture was filtered through a Whatman no. 2 paper. The filtrates were filtered again through a 0.22 µm Millipore filter, before injection into the ion chromatography system.

The ion chromatography system was equipped with: an isocratic pump; conductivity detector; and a model AS-DV auto-sampler (ThermoFisher Scientific, Waltham, MA, USA); a self-generating ERS-500 suppressor (4 mm), a Dionex Ion-Pac AS23 analytical column (4 × 250 mm, particle size 6 µm), and an eluent solution (3.5 mM sodium-carbonate and 1.0 mM sodium-bicarbonate) at a flow rate of 1 mL min^−1^ (Dionex-ThermoFisher Scientific, Waltham, MA, USA) (specifically for anion analysis); a self-generating DRS-600 suppressor (4 mm), a Dionex IonPack CS12A analytical column (4 × 250 mm, 5 µm), and an eluent solution (20 mM methanesulfonic acid) at a flow rate of 1 mL min^−1^ (Dionex-ThermoFisher Scientific, Waltham, MA, USA) (specifically for cation analysis).

#### 2.3.3. Starch and Simple Carbohydrates

Total starch was analyzed using the Megazyme Total Starch Assay kit (AOAC Method 996.11 and AACC Method 76.13; Megazyme International Ireland Ltd., Wicklow, Ireland) based on the enzymatic hydrolysis method, following McCleary et al. [12]. Lyophilized samples (0.03 g) were pre-extracted with 10 mL of 80% ethanol to remove free glucose; the complete starch hydrolization to glucose was achieved by a combined action of α-amylase and amyloglucosidase; the reaction took place by heating up (80 °C) the samples in the presence of an 8 mL sodium acetate buffer (1.2 M) at pH 3.8 and 0.1 mL of thermostable α-amylase (3000 U/mL) for 15 min. After cooling to 50 °C, 0.1 mL of amyloglucosidase (3300 U/mL) was added which catalyzes complete hydrolysis of the maltodextrins to glucose within 30 min. 

The starch concentration was determined as liberated glucose. Then, the mixture was centrifuged at 13,000 rpm (10 min); the supernatant was collected and filtered through a membrane filter (0.45 µm).

Samples were analyzed according to Rohrer [13] using the ICS 3000 System (Dionex-ThermoFisher Scientific, Waltham, MA, USA) and high-performance anion exchange chromatography with pulsed amperometric detection (ED50; Dionex-ThermoFisher Scientific, Waltham, MA, USA), equipped with a Carbopac PA-1 column (CarboPac PA1 Analytical, 4 × 250 mm; particle size 10 µm) (Dionex-ThermoFisher Scientific, Waltham, MA, USA), maintained at 30 °C. Glucose was eluted with NaOH (150 mM) at a flow rate of 1.0 mL min^−1^ for 15 min. Glucose was identified by a comparison of the retention times with the standard. Peak areas were analyzed using the Dionex Chromeleon software (version 6.80, Dionex-ThermoFisher Scientific, Waltham, MA USA).

Total simple carbohydrates were extracted from 15–30 mg of a lyophilized sample and adding 15 mL of ultrapure water and using shaking water baths (Foss, Padova, Italy) for 45 min at room temperature. Then, the mixture was centrifuged at 13,000 rpm (10 min); the supernatant was collected, filtered through a 0.45 µm membrane filter, and analyzed as previously described. 

Carbohydrates were identified by a comparison of the retention times with those of sugar standards. Peak areas were analyzed using the Dionex Chromeleon software (version 6.80, Dionex-ThermoFisher Scientific, Waltham, MA, USA).

#### 2.3.4. Organic Acids

The lyophilized sample (0.3 g) was placed in a 50 mL tube and added to 20 mL metaphosphoric acid (0.1%). The sample was mixed in a shaking water bath at room temperature for 15 min, then centrifuged (12,000 rpm, 4 °C for 15 min) (SR16L, ThermoFisher Scientific, Waltham, MA, USA). The supernatant was collected, filtered, and stored at 4 °C until analyzed according to González-Castro et al. [14] with some modification.

Organic acids were separated by the ICS 3000 HPLC System (Dionex-ThermoFisher Scientific, Waltham, MA, USA) equipped with: An isocratic pump, a 10 µL injection loop, an AS-DV auto-sampler, Hydro-RP 80A column (250 × 4.60 mm) (Phenomenex Inc., Castel Maggiore, BO, Italy), maintained at 30 °C combined with a UV-visible detector (RLSC Diode Array Detector, Dionex-ThermoFisher Scientific, Waltham, MA, USA), set to a wavelength of 210 nm and the Chromeleon version 6.50 software.

The eluent consisted of 100 mM Na_2_SO_4_ at pH 2.6 adjusted with methanesulfonic acid at a flow rate of 1 mL min^−1^. Individual organic acids were identified by comparing retention times with those of available standards.

#### 2.3.5. Volatile Compounds

Volatile compounds were extracted by the HS-SPME technique using a DVB-CAR-PDMS fiber (Supelco, Bellefonte, Pa., USA) according to Gambacorta et al. [15], with some modifications. The freeze-dried powder sample (0.5 g) and 10 μL of internal standard (2-heptanone) in water at 8.15 μg mL^−1^ were placed in a 12 mL screw-cap vial, tightly capped with a PTFE-silicon septum, and conditioned for 10 min at 50 °C. Then, the fiber was introduced into the headspace of the vial for 30 min, removed, and inserted into the gas-chromatography injection port. Desorption of volatiles from the fiber took place in a split mode (1:20 ratio) at 220 °C. The separation of volatile compounds was performed by a Trace 1300 gas chromatograph (Dionex-ThermoFisher Scientific, Waltham, MA, USA) equipped with a VF-WAXms capillary column (Agilent, Santa Clara, CA, USA), 20 m length × 0.10 mm ID x 0.1 μm film, and coupled with an ISQ single quadrupole mass spectrometer (ThermoFisher Scientific, Waltham, MA, USA). The chromatographic conditions were: Oven, 50 °C (0.1 min) to 180 °C at 13 °C min^−1^, to 220 °C at 18 °C min^−1^, held for 3 min; detector, source temperature 250 °C; transfer line temperature 250 °C; carrier gas, helium at a constant flow of 0.4 mL min^−1^. The impact energy was 70 eV. Data were acquired using the full-scan mode in the range of 33 to 280 m/z at an acquisition rate of 7.2 Hz.

Volatiles were identified by comparing the experimental spectra with those obtained by the available pure standard compounds and with those reported in the NIST Library [16] and quantified using relative areas that related the 3-pentanone as an internal standard. The acquisition and processing of peaks were carried out using the Xcalibur v 2.0 software (ThermoFisher Scientific, Waltham, MA, USA).

#### 2.3.6. Phenolic Compounds

The lyophilized sample (0.05 g) was placed in a 2 mL Eppendorf tube and added to 1 mL of 80% methanol in water. The sample was mixed for 1 min, sonicated for 5 min, and then centrifuged (4000 g 4 °C for 20 min). The clear supernatant was diluted 1:1 with an acetonitrile:water (10:90, vol/vol) solvent mixture containing 0.1% formic, and filtered using re-generated cellulose filters of 0.22 μm pore diameter. The analysis was performed according to Pasqualone et al. [17] with some modification using the UHPLC Dionex Ultimate 3000 RS system (quaternary pump, auto-sampler, column oven, and PDA), coupled by the HESI-II probe with the LTQ Velos Pro ion trap mass spectrometer (Dionex-ThermoFisher Scientific, Waltham, MA, USA). The separation of compounds was performed on a Hypersil GOLD aQ C18 column, 100 mm in length, 2.1 mm ID, and 1.9 μm particle size (Waters, Milford, MA, USA) maintained at 30 °C. A binary mobile phase was used: (A) 0.1% formic acid in water and (B) 0.1% formic acid in acetonitrile, at a constant flow of 0.2 mL/min. The gradient program of solvent B was as follows: 0–30 min from 10% to 70%, 30–33 min isocratic 70%, 33–33.1 min from 70% to 10%. The MS conditions were: Capillary temperature 320 °C; source heater temperature 280 °C; nebulizer gas N_2_; sheath gas flow 30 arbitrary units; auxiliary gas flow 15 arbitrary units; capillary voltage—2.8 kV, S-Lens RF Level 60%. Data were acquired in a negative ionization mode.

Phenolic compounds were identified by comparing elution times, molecular ions, MS/MS fragmentation patterns of the experimental spectra with those obtained by the available pure standard compounds or by tentative methods using reported data from the literature. Calibration curves were created to obtain quantification results and were based on the UV signal of each available standard. When no commercial standard was available, a similar compound from the same phenolic group was used as a standard.

### 2.4. Statistical Analysis

A one-way analysis of variance was performed using the Statistical Analysis Software (SAS, Cary, NC, USA). The least significant difference (LSD) test (*p* = 0.05) was used to establish differences between means. 

For a visual analysis of data, the Principal Component Analysis (PCA) was performed using the PAST3 Software (http://folk.uio.no/ohammer/past) [18] on mean standardized ((x-mean)/standard deviation) data. The data matrix considered all genotypes with relative replications. To avoid the presence of highly correlated variables, data were subjected, before the PCA, to the analysis of correlation. The variables which presented a correlation coefficient higher than or equal to 0.8 were grouped, and only one of them was considered in the data matrix.

## 3. Results and Discussion

Refer to Table 1 for acronyms of garlic genotypes.

### 3.1. Bulb Appearance and Biometrical Traits

According to IPGRI descriptors, the Commercial Genotype (CG) used in this study was grouped as a ‘flat globe’ type with a ‘regular multi-cloved radial’ bulb structure. Among the landraces, the bulb shape ranged from ‘broad oval’ for ‘Panni’, ‘PanniD’, and ‘Monteleone’, to ‘flat globe’ for ‘Anzano’ and ‘Cortigli’ and to ‘globe’ for ‘Peschici’. All the landraces studied had an ‘irregular’ bulb structure, except for ‘Peschici’, which was in the ‘regular two-fan group’ (Table S1 and Figure S1).

Concerning the colour of bulbs (with dry tunics) and cloves (with thin skin) (Table S1), all landraces (except for ‘Monteleone’) were characterized by an evident yellow component (+b*) and a very small greenish component (slightly −a*), with a resulting hue and saturation C* values allowing colour identification as creamy. Bulbs and cloves of the ‘Peschici’ landrace distinctively presented a more vivid colour as indicated by the highest C* values. The instrumental data supported the reddish tonality of cloves of the ‘Monteleone’ landrace by the highest +a*, the lowest −b*, along with the highest h° values. 

**Figure 1:**
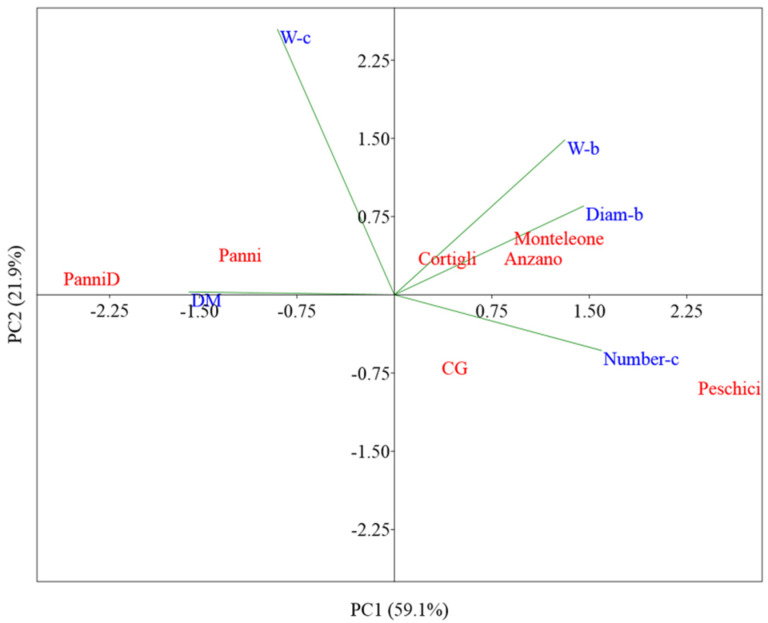
Principal component analysis bi-plot (PC1 vs. PC2) showing the spatial distribution of the main biometrical traits as affected by genotypes. The considered biometrical traits were: Weight of cloves (W-c), number of cloves per bulb (Number-c), weight of bulbs (W-b), diameter of bulbs (Diam-b), concentration of dry matter (DM).

A high variability was observed in biometrical traits of both bulbs and cloves (Table S2). The Principal Component Analysis (PCA), carried out on biometrical data, showed that the first two PCs explained 81% of the total variability, attributing 59.1% to PC1 and 21.9% to PC2 (Figure 1). PC1 discriminates the landraces from the Panni Municipality, clustered on the left-hand side of the axis. ‘PanniD’ was separated from all the other genotypes for the highest concentration in dry matter (DM). The lower moisture concentration of its bulbs is indicative of a higher resistance to post-harvest deterioration. This characteristic may explain the prolonged shelf-life attributed to ‘PanniD’, known by its local name ‘*Aglio Durevole di Panni*’ meaning a long storage life. PCA also discriminates ‘Peschici’ from the other genotypes for the highest number of cloves per bulb (Number-c) (highly and positively correlated to PC1) and by the lowest weight of cloves (W-c) (highly and positively correlated to PC2).

This high variability in morpho-biometrical traits in the examined landraces is, of course, the result of the long process of (un)conscious selection made by farmers and of their continued evolution in a certain eco-geographical area [9]. Although garlic is propagated asexually, using the cloves from the previous growing season, there is a great diversity in morphological (*agronomic*) characters, even between genotypes grown in the same areas for a long time. This is probably mostly a consequence of the accumulation of natural mutations and their evolving over time due to the selection pressures and cultural data in the minds of local farmers [19,20]. 

The variability of the morpho-biometrical traits of garlic genotypes has been widely reported [19,21]. The biometrical traits of the collected landraces are of a high standard in terms of bulb and clove weight in comparison with other Italian landraces [19,22,23] and also in comparison with the tested CG. The DM accumulation of the cloves of collected landraces was also in line with the values reported in various landraces such as those grown in Greece [24] and several genotypes grown in Spain [25].

### 3.2. Mineral Concentration

The concentration of ashes, cations, and anions is reported in Table 2.

The content of ash and cations ranged from the highest values in ‘Peschici’ and ‘Monteleone’ to the lowest in ‘PanniD’. On average, landraces were richer than the CG in ash (51.6 g kg^−1^ dry weight—dw) and cations (15.6 g kg^−1^ dw), particularly K. ‘Cortigli’ along with ‘PanniD’ had the highest Mg concentration, whereas ‘Anzano’ had the highest Ca concentration. Regarding anions, the total concentration in the landraces (1.6 g kg^−1^ dw, on average) was in line with that of the CG; ‘Peschici’ showed the highest values of all the individual anions, followed by ‘Cortigli’ (SO_4_, NO_3_, PO_4_). Among the examined landraces, ‘PanniD’ was the poorest in all anions and cations, except for Mg. 

The genotype and/or environment have been cited to be the main determinants in mineral and ash concentration of garlic bulbs [3,23,24]. Apart from the genotypic characteristics, the high incidence of ash and minerals in ‘Peschici’ in comparison with all the other landraces could also be explained as an effect associated with a higher DM concentration, due to the specific pedo-climatic conditions of this site such as the sandy soils with a limited use of irrigation, and the dry climate (a coastal area characterized by low rainfall and warmer temperatures) (Table 1).

The amount of ash concentration in our landraces (2.0 g 100 g^−1^ fresh weight—fw, on average) was slightly higher than the values reported in Greek garlic landraces [24] and slightly lower than in Indian garlic bulbs [26]. The K levels in our landraces (562 mg 100 g^−1^ fw, on average) can be considered in line with those reported in official food composition databases, such as that of the European Food Safety Authority (EFSA) (579 mg 100 g^−1^ fw) and of the Italian National Centre of Agriculture (INRAN-CREA) (600 mg 100 g^−1^ fw), and higher than those reported by the United States Standard References (USDA) (401 mg 100 g^−1^ fw). The levels of K in our landraces were similar to those found in Greek [24] and in Sicilian (Italy) [23] landraces, while levels of Ca and Mg were significantly lower. 

### 3.3. Starch and Simple Carbohydrates

The values of starch and simple carbohydrates are reported in Table 3. 

Genotypes differed distinctively in the amount of total carbohydrates and individual soluble sugars, except for glucose (0.11 g kg^−1^ dw, on average). ‘Panni’, as well as the CG, showed the highest level of total simple carbohydrates, mainly due to the contribution of sucrose. The CG had the highest value of fructose, followed by ‘Monteleone’ and ‘Anzano’. Since extensive studies on several vegetables have correlated total simple sugars with perceived sweetness [27], among the examined landraces ‘Panni’ had the highest sweet flavour perception. 

On average, the total carbohydrate content in our landraces (1.79 g kg^−1^ dw) was lower than that of the CG (Table 2). Total sugars, sucrose, glucose, and fructose in our landraces were lower than those reported in other landraces from North-Central Italy [21] and Greece [24] and in several genotypes grown in Spain [25]. The value of total sugars in our landraces was also lower than that reported in the INRAN-CREA (8.4 g 100 g^−1^ fw) or the USDA (1.0 g 100 g^−1^ fw) databases.

On average, among the studied landraces, the greatest fraction of simple carbohydrates was represented by sucrose (85%), followed by fructose (10%) and glucose (5%) (Table 2). Sucrose is reported as the main carbohydrate in genotypes collected from various Italian regions [22,28], Spain [25] and Greece [24]. In rare cases, fructose [25] or glucose [29] has been reported as the main sugar in garlic. 

Concerning the starch concentration (Table 2), ‘Anzano’, followed by CG, was the richest and ‘PanniD’ the poorest. On average, the starch content in our landraces (1.44 g kg^−1^ dw; 0.054 g 100 g^−1^ fw) was lower than that of the CG and other genotypes [26,29]. Although starch is generally the most widespread carbohydrate reserve in the plant kingdom, in the examined landraces starch was detected in very low concentrations. Similarly, in other Italian local landraces, only traces of starch have been identified (<0.06 g 100 g^−1^ fw), while fructans have been detected in a larger proportion [10]. Thus, it is possible to suppose that the storage function in the *Allium* spp. bulb tissue could be attributed mainly to fructans.

### 3.4. Organic Acids

The organic acid concentrations of the studied garlic genotypes are presented in Table 3. ‘Monteleone’ along with the CG showed the highest total organic acid concentrations. In the former, a high content of citric, oxalic, and pyruvic acids was detected, while the CG distinctively showed the highest amount of citric acid and the lowest amount of malic and ascorbic acids. ‘Cortigli’, ‘Peschici’, ‘Anzano’, and ‘PanniD’ had the lowest total concentrations of organic acids, and ‘Panni’ had the lowest values of all the individual organic acids, except for malate.

On average, the total organic acid contents in our landraces (25.0 g kg^−1^ dw; 1.0 g 100 g^−1^ fw) were lower than that in the CG (Table 2), and in other genotypes such as those from Greece [24] (2.79 g 100 g^−1^ fw) and from Latvia (3.87 g 100 g^−1^ dw) [30].

In the examined landraces, the organic acid profile was represented by 43% citric, 21% malic, 18% oxalic, and 18% pyruvic acid (Table 2). The organic acid composition of our landraces varied greatly compared with other literature data. A wider composition of organic acids was found in garlic samples from Latvia by Priecina and Karklina [30] and in Italian garlic varieties by Ritota et al. [28], additionally reporting the presence of fumaric, formic, succinic, quinic, salicylic, and tartaric acids, probably due to a different assay implemented.

In the collected landraces, and particularly in ‘Monteleone’, ‘Peschici’, and ‘Anzano’, ascorbic acid (the main biologically active form of vitamin C) was distinctively higher than in the CG (Table 2). However, the averaged values were lower than those reported for some Italian varieties by Fratianni et al. [6] and those reported in the INRAN-CREA (5 mg 100 g^−1^ fw) and in the USDA (31.2 mg 100 g^−1^ fw) food composition databases. 

### 3.5. Volatile Compounds

The volatile compound concentration in the studied garlic genotypes is reported in Table 4.

The precursors of the volatile organic S-compounds in garlic are γ-glutamyl cysteine and cysteine sulfoxides, including deoxyallin and mainly alliin (S-allyl-cysteine-S-oxide) [4], an odourless derivative of cysteine. This latter is enzymatically hydrolyzed (allinase or alliin lyase, E.C.4.4.1.4) into a mixture of both volatile and non-volatile S-containing compounds, after the breakage of the tissue caused by cutting, mastication, and cooking [31]. The volatile S-containing compounds include thiosulphinates, which are very unstable and are transformed into compounds belonging to Sulphides-di-allyl-sulphide (DAS), di-allyl-disulphide (DADS), di-allyl-trisulphide (DATS) or into compounds belonging to ‘Vinyldithiins’ (cyclic sulfur-containing compounds) or into others [31]. All of them are responsible for the typical flavour of garlic and the protective effects against cardiovascular diseases [1,2,32].

In the current study, the main fraction of volatile substances was the S-compounds (94% for landraces; 79% for the CG), with the non-sulphur compounds (including aldehydes, and ‘others’) representing only a minor fraction (6% for landraces; 21% for the CG). Among the examined genotypes, a total of 28 compounds were identified in the volatile fraction, 16 of them were S-compounds, while the other compounds were hydrocarbons, alcohols, and ethers (Figure S2).

The total volatile concentration in CG bulbs was distinctively higher than those of landraces (15.0 vs. 2.2 mg kg^−1^ dw, on average), mainly due to the amount of S-compounds (37.8 vs. 2.1 mg kg^−1^ dw, on average). Among the non-sulphur volatile compounds, a lower concentration of total aldehydes (0.04 mg kg^−1^ dw, on average) and ‘other’ compounds (0.10 mg kg^−1^ dw, on average) was found in the examined landraces. In particular, the landraces were completely devoid of two out of four of the determined aldehydes—‘2-butenal-2-methyl’ and ‘4-heptenal’. 

Di-methyl-sulphide, trans-propenyl-methyl-di-sulphide, mercaptoacetic acid, 3-vinyl-4H-1,2-dithiin, and Allicin (S-oxo-di-allyl-di-sulphide) (among the S-compounds), and 4-heptenal-enantic aldehyde (among aldehydes), were detected in very low amounts (<0.01 unit).

Except for the 1-propanethiol S-compound, the genotype distinctively affected the concentration and the profile of volatile compounds.

A large variation in the amount of (S)-volatile compounds was observed between the GC and the collected landraces (15.0 vs. 2.2 mg kg^−1^ dw—total volatile compounds; 12.0 vs. 2.0 mg kg^−1^ dw—S-compounds). Apart from the genotype, other factors such as ‘environment’ may affect volatile compound concentrations, as widely proved in the specific literature on garlic [1,33]. The large difference in concentration of (S)-volatile compounds between the CG and landraces could be imputable to different pre- and/or post-harvest management. It is well known that (S) fertilization is strictly correlated to the accumulation of garlic volatile S-compounds [34] and of organo-sulfur-compounds [35], precursors of volatile S-compounds [36]. The collected landraces might have accumulated lower (S) volatile compounds, probably due to the poor S availability in the marginal soils where these plants are collected, also characterized by a low organic matter content and no fertilizer application (in normal growing conditions various fertilizers are used containing significant amounts of sulfur, thus improving S availability). The different post-harvest storage time between the CG and landraces could have also affected their volatile (S)-compound contents. Several studies report that the concentrations of volatile [37] and non-volatile [38] S-compounds are higher in stored than in fresh material. The total amount of volatile (S)-compounds was lower than that found in other studies [4,39]. The allicin in our landraces, representing less than 1% of the total volatile compounds and of the S-compounds, was slightly lower than that found in five endemic Italian varieties [6] and much lower than that found in red garlic from Argentinian germplasm [40] and in Croatian garlic [8].

The qualitative profile of the volatile component in the current study was instead in line with that found in numerous studies reported in the garlic literature [4,32,37,39,41,42]. Genotype and/or ‘environment’ are also the main determinants of the composition of volatile compounds [1,33].

In the current study, in the examined landraces the five most abundant S-compounds (representing 93% of the S-compounds) were ‘di-allyl-trisulphide’ (DATS) (0.67 mg kg^−1^ dw; 32% of S-compounds), followed by 3-vinyl-1,2-dithyocyclohex-5-en’ (0.47 mg kg^−1^ dw; 23% of S-compounds), di-allyl-disulphide (DADS) (0.36 mg kg^−1^ dw; 18%), 3-vinyl-1,2-dithiin (0.32 mg kg^−1^ dw; 15%), and methyl-allyl-tri-sulphide (0.10 mg kg^−1^ dw; 5%). The quantitative analysis in the CG showed that five compounds represented 85% of the total S-compounds: 3-vinyl-1,2-dithyocyclohex-5-ene (40% of the total), 3-vinyl-1,2-dithiin (18%), DATS (10%), and DADS (8%) and methyl-2-propenyl-di-sulphide (8%).

The sulphide S-compounds were prevalent in the collected landraces, while the S-compounds belonging to the ‘Vinyldithiins’ family were prevalent in the CG. Although initially controversial, the S-compounds belonging to the ‘Vinyldithiin’ family are now considered the major components of fresh garlic and some of them have been found to have a very high flavour dilution factor (FD ≥ 1) indicating an intense pungent odour [32,43]. Accordingly, the CG should have a more pungent aroma than the collected landraces. It can be not excluded that the examined landraces were selected over the time as a conscious and intentional aim by growers to reduce the volatile (S)-compounds, responsible for breath and sweat smelling of garlic, which can last for days. Thus, landraces showed this drawback to a more limited extent.

### 3.6. Phenolic Compounds

Phenolic compounds are reported in Table 5. In the examined genotypes, the phenolic acids (six compounds) represented most of the total phenols (83% on average), and among them, ferulic acid (524 mg kg^−1^ dw, on average) was the most abundant, followed by the caffeic acid derivates—caffeic acid-O-hexoside-1 (211 mg kg^−1^ dw, on average) and caffeic acid-O-hexoside-2 (203 mg kg^−1^ dw, on average). The flavonoid component (six compounds), represented 16% of the total phenols, and among them, iso-rhamnetin (120 mg kg^−1^ dw, on average) and rutoside-1 (60 mg kg^−1^ dw, on average) were the most abundant. Less than 0.3% of total phenols were unknown compounds. A 3-hydroxy-methyl phenol was found only in the CG. Among the flavonoids, quercetol (quercetin), patuletin, hyperoside (hyperin), iso-quercitrin (quercetin 3-β-D-glucoside), and coumaroyl-quinic acid among the phenolic acids were detected at a very low rate (<0.5 unit). 

Except for caftaric and caffeic acid-O-hexoside-1 acids, the genotype distinctively affected the concentrations and the profile of phenols. 

On average, the phenolic concentration in our landraces was much higher than in the CG (1230 vs. 533 mg kg^−1^ dw), due to the high contribution of both components. The CG showed a very small amount of flavonoids (only quercetol). 

The high level of phenols in all landraces in comparison with the CG, could be explained as an effect associated with the genotype, but also with the environment [1], the pedo-climatic conditions of the growing site and the technical practices adopted by growers. It is well known that the biosynthesis of phenols/flavonoids is upregulated in response to a wide range of abiotic stresses/factors, ranging from nutrient depletion to cold and drought stress [44,45], aiming to effectively counter the stress-induced oxidative damage. Thus, apart from the genotypic characteristics, the incidence of phenols in the landraces could be related to harsh growing conditions in terms of climate and/or to the scarce availability of nutrients/water, since no or low inputs (water, nutrients, chemicals) were provided by the landrace growers.

The levels of phenolic compounds in our landraces were also substantially greater than that reported for Polish garlic (11.5 mg kg^−1^ dw) [7] and particularly in phenolic acids (0.1–252.1 mg kg^−1^ dw) [46], as well as for the local garlic from Namhae-gun (Korea) both in total phenolic acids (17.9 mg kg^−1^ dw) and in flavonoids (29.9 mg kg^−1^ dw) [47].

The antioxidant, antibacterial, anti-inflammatory, anti-proliferative, and chemopreventive properties of phenolics and flavonoids in vegetables and Allium spp. [2,48] are well known, thus the contribution of the observed landraces to the protection and preservation of human health should be emphasized due to their being very rich in these compounds.

Among the examined landraces, ‘Peschici’ and ‘Monteleone’, followed by ‘Cortigli’ and ‘Anzano’ showed the highest total phenol concentrations. The most abundant compounds were ferulic acid along with caffeic acid-O-hexoside-2 (phenolic acids) and iso-rhamnetin and rutinoside-1 (flavonoid compounds). In this study, we found significantly higher amounts of ferulic acid both in comparison with the tested GC and also compared with those found in several genotypes in Spain −3.5 mg kg^−1^ dw in Almeria [49]; 0.9–7.3 mg kg^−1^ dw in Andalusia [50]; and in Poland −0.3 mg kg^−1^ dw [7]. Ferulic acid, conferring rigidity to cell walls [51], has been identified as being involved in resistance against thrips [52], stem borers in maize and cotton, and cereal aphids and midges, and in defence against different fungi [53]. Therefore, the higher presence of this compound might be linked to a defence mechanism of landraces to biotic factors.

Hydroxycinnamic acids as well as ferulic and caffeic acids have been reported, similar to many phenols, as antioxidants since they are reactive toward free radicals as a reactive oxygen species [54]. Caffeic acids, found in coffee, fruit, and vegetables such as garlic, are a well-known pharmacological antioxidant with anti-mutagenic activities and anti-inflammatory and immune-modulatory effects. They are also an anti-wrinkle agent and inhibitor of carcinogenesis, as reported by Kim et al. [47]. Moreover, according to the findings of Kallel et al. [55], ferulic and caffeic acids may be the main compounds responsible for the antimicrobial effect of the crude garlic extract.

Concerning iso-rhamnetin, it was identified as a new flavonoid glycoside in *Allium neapolitanum* and evaluated for its anti-aggregation human platelet activity by Carotenuto et al. [56]. Recently, pre-treatment with iso-rhamnetin, extracted from sea buckthorn (*Hippophae rhamnoides* L.), has been shown to play a protective role against acute fulminant hepatitis in mice [57].

‘Peschici’ and ‘Monteleone’ appreciably highlighted a high amount of rutoside-1, known as rutin (a flavonol glycoside between quercetin and disaccharide rutinose), which has been found to have important pharmacological effects, such as in the prevention of ulcerative colitis [58]. Both ‘Peschici’ and ‘Monteleone’ also showed the highest amounts of iso-quercitrin (also named quercetin 3-β-D-glucoside), even if identified at low concentrations, this quercetin glucoside, which can be isolated from several *Allium* spp. (Chinese onion, garlic, onion, and Welsh onion), has been shown to have anti-proliferative potential in various cancer cell lines [48].

‘Anzano’ along with ‘Monteleone’ were the only two genotypes containing patuletin, a compound also detected on *Allium* spp. from Romania (A. *obliquum* L.; A. *senescens* L. subsp. *montanum* (Fries) Holub; A. *schoenoprasum* L. subsp. *Schoenoprasum*) [59]. Isolated in *Urtica urens* L., patuletin has been shown to possess an antioxidant activity and free radical scavenging effects in rats treated with aflatoxin B1, a risk factor for hepatocellular carcinoma [60]. Furthermore, its anti-proliferative, necrotic, and apoptotic activity in tumour cell lines has been claimed recently by Alvarado-Sansininea et al. [61].

Among the examined landraces, those from Panni Municipality were the poorest in total phenolic compounds but contained a good level of caffeic acid-O-hexoside-1 acid (phenolic acid) and iso-rhamnetin and rutoside-1 (flavonoids). Among the investigated genotypes, ‘PanniD’ showed the highest value of hyperoside (hyperin) and it was the only landrace which accumulated a considerable amount of quercetol (quercetin). Quercetol (quercetin) has important functional benefits, including an anti-inflammatory activity, an anti-histamine effect, allergy medication, as well as anticancer and antivirus activities. It has also been claimed to regulate blood pressure in hypertensive subjects [62]. The hyperoside (hyperin) flavonoid has also been detected in other common Italian varieties of garlic [6]. Its ROS scavenging activity is well known and are the protective effects for PC12 cells against induced-cytotoxicity [63]. This flavonoid is also a candidate as a therapeutic agent for the treatment of vascular inflammatory diseases in humans and in mice endothelial cells [64].

### 3.7. Principal Component Analysis (PCA)

**Figure 2:**
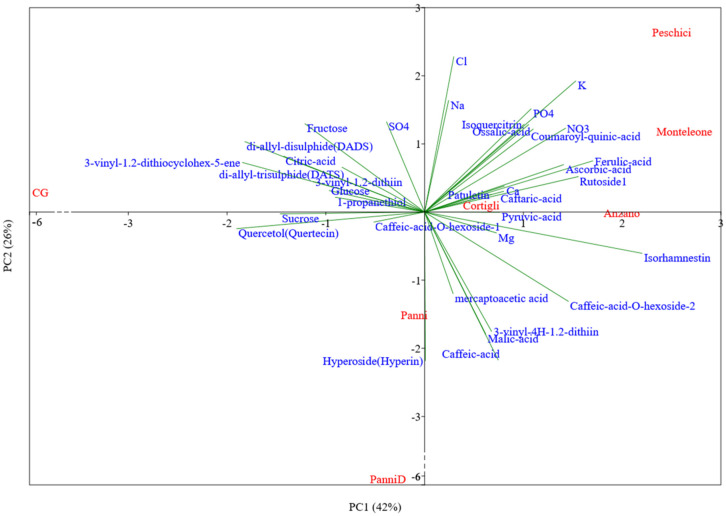
Principal component analysis bi-plot (PC1 vs. PC2) showing the spatial distribution of the main chemical traits as affected by genotypes. Refer to Table 1 for the acronyms of garlic genotypes. Following a previous analysis of correlation, 3-vinyl-1.2-dithiocyclohex-5-ene is representative of a group of seven volatile S-compounds (di-methyl-sulphide, propylene-sulphide, methyl-2-propenyl-disulphide, trans-propenyl-methyl-disulphide, di-methyl-trisulphide, methyl-allyl-trisulphide, and Allicin-S-oxo-di-allyl-di-sulphide); di-allyl-disulphide (DADS) is representative of a group of two volatile S-compounds (di-allyl-sulphide-DAS and 2-vinyl-1.3-dithiane); Isorhamnetin is representative of 3-hydroxy-methyl-phenol.

The results of the PCA using the chemical parameters showed that the first two PCs explained approximately 70% of the total variability, attributing 42% to PC1 and 26% to PC2 (Figure 2). 

The group of ‘Anzano’, ‘Monteleone’, ‘Cortigli’, and ‘Peschici’ landraces separated from the ‘PanniD’, ‘Panni’ landraces (low side of PC2) and also from the CG (left side of PC1). 

The former group clustered for the concentration of phenolic compounds (Isorhamnetin, Ferulic acid Caffeic-acid-O-hexoside-2, and Rutoside1) (highly and positively correlated to PC1) and for the high concentration of anions and cations (highly and positively correlated to PC2). ‘Peschici’ and ‘Monteleone’ set in an extreme position of this quadrant, mainly characterized by the highest mineral (K) concentration and phenols, as also underlined by Anova (Table 2 and Table 5). ‘Anzano’ and ‘Cortigli’ clustered quite in a cloud-graphical space, suggesting a substantial homogeneity in chemical composition. 

Conversely, the CG tended to cluster separately on the left side of PC1 for the general negative correlation with phenolic compounds and for the positive correlation with the volatile S-compounds (di-allyl-disulphide-DADS and 3-vinyl-1,2-dithiocyclohex-5-ene), with a phenolic compound as Quercetol (Quertecin) (exclusively present in GC, Table 5), citric acid and sucrose.

The PC2 axis separated the two Panni Municipality landraces from all the other landraces mainly for the general negative correlation with all the minerals and phenols, except for Hyperoside (Hyperin), exclusively present in ‘PanniD’ (Table 5).

## 4. Conclusions

The observed diversity in the morpho-biometrical and chemical traits among the examined landraces can be explained by considering the long process of adaptation to the specific ‘environment’ (pedo-climatic properties and less intensive agronomic practices), along with the constant selection pressure performed by farmers over a long period. These factors ensured a ‘non-homologation’ of the final product and specificity of the morpho-biometrical and chemical characteristics of a single genotype. Findings highlighted that the landraces collected from the province of Foggia maintain high biometrical (*productive*) standard features for the market, in line with those of the examined commercial genotype used as a reference standard.

The examined landraces were able to accumulate nutrients and phytochemicals, thus showing interesting features for the human diet. They exhibited high total cations (mainly K^+^) and phenols (ferulic acid and derivates of caffeic, among phenolic acids, iso-rhamnetin and rutinoside-1, among flavonoids). 

By increasing the knowledge of the properties of these local garlic landraces, this work could represent a key tool for the promotion of their consumption. At the same time, the consumption could encourage their cultivation, thus contributing to the protection of this agro-biodiversity at a high risk of genetic erosion by promoting their in situ/on-farm conservation.

## Figures and Tables

**Table 1:** List of garlic genotypes and description of collecting areas.

Genotype	Acronym	Collection Sites	Landscape Unit ^1^	Latitude (N)	Longitude (E)	Altitude (m, asl)	Yearly Mean Temperature ^2^ (°C)	Yearly Mean Rainfall ^2^ (mm)
Spanish white garlic (Commercial genotype)	‘CG’	Grocery store						
Aglio dei Cortigli (Landrace)	‘Cortigli’	Vico del Gargano (Municipality) ‘Cortigli’ locality	Gargano (mountain)	41.8960	15.9596	445	11.9 ^3^	721 ^3^
Aglio di Peschici (Landrace)	‘Peschici’	Peschici (Municipality)	Gargano (Adriatic Sea coastal plain)	41.94711	16.0098	12	16.6	459
Aglio Rosso di Monteleone di Puglia (Landrace)	‘Monteleone’	Monteleone di Puglia (Municipality)	Daunian Apennine (mountain)	41.16768	15.2418	800	11.7	572
Aglio di Anzano di Puglia (Landrace)	‘Anzano’	Anzano di Puglia (Municipality)	Daunian Apennine (mountain)	41.12126	15.2914	760	11.6	571
Aglio Bianco di Panni (Landrace)	‘Panni’	Panni (Municipality)	Daunian Apennine (mountain)	41.22080	15.2762	728	12.1	558
Aglio Durevole di Panni (Landrace)	‘PanniD’	Panni (Municipality)	Daunian Apennine (mountain)	41.22080	15.2762	729	12.1	558

^1^ As reported in Piano Paesaggistico Territoriale Regionale—PPTR—www.paesaggiopuglia.it/pptr. ^2^ As reported in https://it.climate-data.org/. ^3^ As reported in http://my.meteonetwork.it/station/.

**Table 2:** Concentration of ashes and inorganic ions (g kg^−1^ dw) in bulbs of garlic genotypes.

Genotype ^1^	Ashes	Inorganic Cations					Inorganic Anions				
		Total	Na	K	Mg	Ca	Total	NO_3_	Cl	PO_4_	SO_4_
‘CG’	34.1 e^3^	8.93 d	0.74 a	7.81 d	0.06 d	0.32 c	1.609 c^3^	0.000 c	0.707 b	0.200 c	0.702 b
‘Cortigli’	55.0 b	15.9 b	0.68 a	14.5 b	0.14 a	0.58 b	1.963 b	0.094 b	0.618 c	0.419 b	0.833 a
‘Peschici’	70.0 a	22.0 a	0.76 a	20.5 a	0.11 b	0.55 b	2.766 a	0.140 a	1.130 a	0.633 a	0.864 a
‘Monteleone’	68.7 a	21.7 a	0.89 a	20.4 a	0.11 b	0.37 c	1.452 c	0.081 b	0.627 bc	0.366 b	0.379 d
‘Anzano’	49.4 c	13.8 c	0.72 a	12.0 c	0.07 cd	1.09 a	1.651 c	0.143 a	0.602 c	0.380 b	0.527 c
‘Panni’	42.7 d	14.2 c	0.44 b	13.1 c	0.09 bc	0.61 b	1.351 d	0.030 c	0.588 c	0.225 c	0.508 c
‘PanniD’	23.7 f	5.90e	0.30 b	5.22 e	0.12 ab	0.26 c	0.732 e	0.014 c	0.327 d	0.161 c	0.231 e
Significance ^2^	***	***	***	***	***	***	***	***	***	***	***

^1^ Refer to Table 1 for acronyms of garlic genotypes. ^2^ *** Significant at *p* ≤ 0.001. ^3^ Different letters within the column indicate significant differences at *p* = 0.05.

**Table 3:** Concentration of starch, simple carbohydrates, and organic acids (g kg^−1^ dw) in bulbs of garlic genotypes.

Genotype ^1^	Starch	Simple Carbohydrates				Organic Acids					
		Total	Glucose	Sucrose	Fructose	Total	Oxalic	Citric	Pyruvic	Malic	Ascorbic
‘CG’	2.14 b^3^	6.22 a	0.18 a	5.59 b	0.45 a	32.4 ab^3^	2.94 bc	26.5 a	1.96 c	0.94 e	<0.001 c
‘Cortigli’	0.83 de	1.28 b	0.19 a	0.84 c	0.25 ab	19.6 d	4.00 b	9.52 c	3.10 c	2.91 de	0.02 ab
‘Peschici’	1.30 cd	0.54 b	0.06 a	0.33 c	0.15 b	19.1 d	4.45 b	7.51 c	3.88 bc	3.26 cde	0.02 a
‘Monteleone’	1.78 bc	0.57 b	0.07 a	0.29 c	0.21 ab	40.7 a	9.09 a	18.7 b	7.09 a	5.77 bc	0.03 a
‘Anzano’	3.06 a	0.47 b	0.10 a	0.15 c	0.22 ab	17.8 d	4.25 b	6.60 c	3.19 c	3.72 cd	0.02 a
‘Panni’	1.23 d	7.38 a	0.10 a	7.13 a	0.15 b	30.2 bc	3.32 bc	13.3 bc	6.38 ab	7.20 ab	0.01 bc
‘PanniD’	0.46 e	0.50 b	0.07 a	0.37 c	0.06 b	22.5 cd	1.88 c	8.62 c	3.17 c	8.85 a	0.01 bc
Significance ^2^	***	***	ns	***	**	***	***	***	***	***	***

^1^ Refer to Table 1 for acronyms of garlic genotypes. ^2^ Significance—ns and **, *** not significant or significant at *p* ≤ 0.01 and *p* ≤ 0.001, respectively. ^3^ Different letters within the column indicate significant differences at *p* = 0.05.

**Table 4:** Profile of volatile compounds (mg kg^−1^ dw) in bulbs of garlic genotypes.

RT ^1^	Volatile Compounds	MW ^2^	*m/z* ions	Genotype ^3^							Signifi- cance ^4^
				CG	Cortigli	Peschici	Monteleone	Anzano	Panni	PanniD	
	***total***			*14.98 a^5^*	*1.72 b*	*3.35 b*	*1.50 b*	*2.11 b*	*1.81 b*	*2.69 b*	***
	***S-compounds***			*11.77 a*	*1.64 b*	*3.12 b*	*1.43 b*	*2.00 b*	*1.70 b*	*2.48 b*	***
1.43	di-methyl-**sulphide** ^6^	62	62,47,35	0.044 a	0.001 b	0.000 b	0.001 b	0.002 b	0.000 b	0.000 b	*
1.71	1-propanethiol	76	76,47,43	0.149 a	0.030 a	0.030 a	0.016 a	0.034 a	0.037 a	0.045 a	ns
2.03	propylene-**sulphide**	74	41,74,46	0.219 a	0.006 b	0.006 b	0.007 b	0.015 b	0.009 b	0.022 b	***
3.57	di-allyl-**sulphide** (DAS) ^6^	114	45,41,73,39	0.316 a	0.009 b	0.023 b	0.014 b	0.010 b	0.012 b	0.021 b	***
4.86	methyl-2-propenyl-**disulphide**	120	120,41,45	0.906 a	0.017 b	0.068 b	0.016 b	0.039 b	0.008 b	0.007 b	***
4.91	trans-propenyl-methyl-**disulphide**	120	73,120,45	0.038 a	0.001 b	0.009 ab	0.001 b	0.006 b	0.001 b	0.005 b	*
5.89	di-methyl-**trisulphide**	126	126,45,79	0.187 a	0.001 b	0.021 b	0.001 b	0.005 b	0.000 b	0.000 b	***
6.69	mercaptoacetic acid	92	47,45,92	0.000 d	0.002 abc	0.001 abcd	0.001b cd	0.000 cd	0.003 a	0.003 ab	*
6.86	di-allyl-**disulphide** (DADS) ^6^	146	41,81,39	0.929 a	0.304 c	0.478 b	0.335 c	0.369 bc	0.281 c	0.402 bc	***
7.93	methyl-allyl-**trisulphide**	152	87,73,45	0.522a	0.054 cd	0.306 b	0.039 d	0.142 c	0.017 d	0.013 d	***
9.27	3-vinyl-1,2-dithiin	144	45,144,97	2.203 a	0.255 b	0.424 b	0.201 b	0.303 b	0.286 b	0.439 b	*
9.49	2-vinyl-1,3-dithiane	146	74,72,45	0.222 a	0.024 b	0.037 b	0.020 b	0.051 b	0.021 b	0.046 b	***
9.73	di-allyl-**trisulphide** (DATS) ^6^	178	73,41,113	1.219 a	0.521 c	1.138 ab	0.461 c	0.528 c	0.575 bc	0.771 abc	*
10.20	3-vinyl-1,2-dithiacyclohex-5-ene	144	72,71,144	4.766 a	0.389 b	0.555 b	0.305 b	0.457 b	0.435 b	0.682 b	***
10.52	3-vinyl-4H-1,2-dithiin	144	72,71,144	0.000 d	0.008 bc	0.002 cd	0.004 bcd	0.021 a	0.010 b	0.017 a	***
10.56	Allicin (S-oxo-di-allyl-**disulphide**)	162	41,45,72	0.049 a	0.011 b	0.022 b	0.007 b	0.019 b	0.009 b	0.009 b	***
	***Non-sulphur compounds***			*3.22 a*	*0.09 b*	*0.24 b*	*0.07 b*	*0.10 b*	*0.10 b*	*0.31 b*	***
	***Aldehydes***			*1.01 a*	*0.01 b*	*0.11 b*	*0.00 b*	*0.01 b*	*0.02 b*	*0.08 b*	***
2.71	2-butenal (crotonaldehyde) ^6^	70	41,39,70	0.564 a	0.009 b	0.107 b	0.000 b	0.013 b	0.017 b	0.072 b	***
3.05	2-butenal-2-methyl (2-methyl-2-pentenoic aldehyde)	84	55,84,29,27	0.248 a	0.000 b	0.000 b	0.000 b	0.000 b	0.000 b	0.000 b	**
3.79	4-heptenal (enantic aldehyde)	112	68,67,55	0.062 a	0.000 b	0.000 b	0.000 b	0.000 b	0.000 b	0.000b	***
5.93	nonanal (pelargonaldehyde) ^6^	142	57,98,43,56	0.185 a	0.000 b	0.002 b	0.001 b	0.001 b	0.001 b	0.004 b	***
	***Others***			*2.20 a*	*0.08 b*	*0.13 b*	*0.07 b*	*0.09 b*	*0.08 b*	*0.14 b*	***
1.28	Cyclopropane	42	42,41,39,27	0.482 a	0.023 b	0.034 b	0.019 b	0.033 b	0.024 b	0.037 b	***
1.38	heptane (di-propyl-methane)	100	43,41,29,57	0.220 a	0.000 b	0.000 b	0.000 b	0.000 b	0.000 b	0.000 b	***
3.20	2-propen-1-ol (allyl-alcohol)	58	57,29,31	0.438 a	0.021 b	0.037 b	0.019 b	0.029 b	0.023 b	0.032 b	***
5.11	Pentadecane	212	57,43,71	0.124 a	0.002 b	0.001 b	0.004 b	0.001 b	0.005 b	0.004 b	*
5.33	di-iso-deciyl-ether	298	43,57,41	0.230 a	0.003 b	0.008 b	0.003 b	0.002 b	0.003 b	0.009 b	***
6.07	tetradecane	198	57,43,71	0.083 a	0.003 b	0.015 b	0.002 b	0.000 b	0.002 b	0.010 b	**
6.29	benzene,m-ditert-butyl	190	175,57,41	0.077 a	0.004 b	0.008 b	0.003 b	0.000 b	0.004 b	0.011 b	**
7.33	1,4-dihydro-2,3-benzoxathin-3-oxide	168	104,103,105	0.551 a	0.020 b	0.025 b	0.015 b	0.026 b	0.022 b	0.033 b	***

^1^ Retention time (min). ^2^ Molecular weight. ^3^ Refer to Table 1 for acronyms of garlic genotypes. ^4^ Significance—ns and *, **, *** not significant or significant at *p* ≤ 0.05, *p* ≤ 0.01, *p* ≤ 0.001, respectively. ^5^ Different letters within the row indicate significant differences at *p* = 0.05. ^6^ Comparison with standards.

**Table 5:** Phenolic compounds (mg kg^−1^ dw) in the bulb of garlic genotypes.

Phenols	RT (min)	UV Max (nm)	[M-H]^−^	m/z ions	Genotype ^1^							Signifi- cance ^2^
					CG	Cortigli	Peschici	Monteleone	Anzano	Panni	PanniD	
Caffeic acid-O-hexoside-1 ^5^	1.58	379	341	179, 135	404.1 a^3^	186.9 a	181.5 a	141.8 a	169.7 a	192.8 a	205.3 a	ns
Caffeic acid-O-hexoside-2 ^5^	1.90	379	341	179, 135	119.6 b	220.0 a	231.8 a	187.8 a	196.0 a	230.3 a	235.2 a	*
Caffeic acid ^4^	2.18	323	179	135, 107	6.81 d	10.8 c	5.61 d	14.3 b	14.4 b	10.7 c	17.8 a	***
Ferulic acid ^5^	2.74	320	195	177, 89	87.6 d	518.6 bc	851.4 a	790.9 ab	611.6 abc	405.6 c	404.8 c	**
Caftaric acid ^4^	3.49	308	311	179	1.87 a	14.7 a	12.2 a	30.2 a	29.0 a	30.5 a	0.00 a	ns
Coumaroylquinic acid ^5^	11.33	306	337	191, 163	0.00 b	0.17 b	0.73 b	0.00 b	0.68 a	0.00 b	0.00 b	***
Rutoside-1 (Rutin) ^4^	9.21	354	609	609, 300	0.00 b	68.3 ab	91.2 a	102.1 a	68.1 ab	38.3 ab	50.3 ab	*
Iso-rhamnetin ^6^	10.05	374	315	165	0.00 b	137.8 a	157.9 a	142.3 a	139.7 a	126.7 a	132.3 a	***
Hyperoside (Hyperin) ^4^	10.20	355	463	463, 300	0.00 b	0.00 b	0.20 b	0.00 b	0.00 b	0.22 b	1.09 a	**
Iso-quercitrin (Quercetin 3-ß-d-glucoside) ^4^	10.77	355	463	463, 300	0.00 b	0.17 b	0.92 ab	1.39 a	0.00 b	0.00 b	0.00 b	*
Patuletin ^6^	12.97	374	331	151	0.00 b	0.00 b	0.00 b	0.20 ab	0.43 a	0.00 b	0.00 b	*
Quercetol (Quercetin) ^4^	14.22	374	301	151, 301	0.49 a	0.00 c	0.00	0.00 c	0.00 c	0.00 c	0.22 b	***
3-Hydroxy-methyl phenol ^5^	20.03	280	123	123	1.94 a	0.00 b	0.00 b	0.00 b	0.00 b	0.00 b	0.00 b	***
Sum of un-identified phenols					4.2 c	4.4 c	3.0 cd	1.0 e	6.7 b	2.0 de	8.8 a	***
Phenolic acids					526.5 c	954.3 ab	1193 a	1128 ab	1030.3 ab	881.2 b	888.8 b	**
Flavonoids					0.49 d	203.8 bc	248.0 ab	271.1 a	207.5 abc	155.7 c	189.7 bc	***
Total phenols					532.8 c	1162 ab	1444 a	1400 a	1,245 ab	1039 b	1087 b	***

^1^ Refer to Table 1 for acronyms of garlic genotypes. ^2^ Significance—ns, *, **, and *** not significant or significant at *p* ≤ 0.05, *p* ≤ 0.01, and *p* ≤ 0.001, respectively. ^3^ Different letters within the row of samples indicate significant differences at *p* = 0.05. ^4^ Comparison with standards. ^5^ Comparison with the caffeic acid standard. ^6^ Comparison with the quercetol standard.

## Supplementary Materials

The following are available online at https://www.mdpi.com/2304-8158/9/7/850/s1. Figure S1: Photos of the bulbs of the studied garlic genotypes. Figure S2: Examples of HS-SPME chromatograms of volatile compounds. Table S1: Morphological traits of bulbs and cloves of garlic genotypes. Table S2: Biometric traits of bulbs and cloves of garlic genotypes.
