# Supplementary material for: Evaluation of Garlic Landraces from Foggia Province (Puglia Region; Italy)
Source: Foods. 2020 Jun 29;9(7):850. doi: 10.3390/foods9070850 (PMC7404550; doi:10.3390/foods9070850)
Supplement: Supplementary file 1 [file foods-09-00850-s001.zip › Table S1.docx]

Table 1. Morphological traits of bulbs and cloves of garlic genotypes.

| Genotype | Acronym | Bulb  Shape^2^  (7.1.11) | Bulb Structure type^2^  (7.1.20) | Bulb  colour^2^  (7.1.16.1) | Clove  colour^2^  (7.1.16.2) | Bulb (with dry tunics)^3^ | | | | | Clove (with thin skin)^3^ | | | | |
| --- | --- | --- | --- | --- | --- | --- | --- | --- | --- | --- | --- | --- | --- | --- | --- |
|  |  |  |  |  |  | L* | h° | C* | a* | b* | L* | h° | C* | a* | b* |
| Spanish white garlic  (Commercial genotype) | ‘CG’ | 2 | 1 | 1 | 1 | 85.8 a^1^ | 107.3 b | 2.6 c | -0.7 a | 2.5 c | 88.9 a | 105.5 bc | 4.2 e | -1.1 c | 4.1 e |
| Aglio dei Cortigli  (Landraces) | 'Cortigli' | 2 | 6 | 2-3 | 1-2 | 76.5 c | 98.7 c | 8.3 b | -1.3 ab | 8.2 b | 75.5 c | 101.0 bc | 15.2 b | -2.9 c | 14.9 b |
| Aglio di Peschici  (Landraces) | 'Peschici' | 5 | 2 | 2-3 | 1-2 | 75.2 c | 99.7 c | 13.2 a | -2.2 c | 12.9 c | 76.0 bc | 99.4 bc | 20.7 a | -3.4 c | 20.4 a |
| Aglio Rosso di Monteleone di Puglia  (Landraces) | 'Monteleone' | 4 | 6 | 2-5 | 2-4 | 81.5 b | 135.9 a | 5.2 bc | -3.7 d | 3.6 c | 79.6 b | 349.1 a | 14.2 bc | 14.0 a | -2.1 f |
| Aglio di Anzano di Puglia  (Landraces) | 'Anzano' | 2 | 6 | 2-3 | 1-2 | 85.7 a | 107.9 b | 4.7 c | -1.6 abc | 4.4 c | 84.8 a | 98.3 c | 12.5 bcd | -1.8 c | 12.3 bc |
| Aglio Bianco di Panni  (Landraces) | 'Panni' | 4 | 6 | 2-3 | 1-2 | 75.8 c | 97.2 c | 14.5 a | -1.7 bc | 14.4 a | 67.7 d | 80.2 d | 11.3 cd | 1.8 b | 11.0 cd |
| Aglio Durevole di Panni  (Landraces) | 'PanniD' | 4 | 6 | 2-3 | 1-2 | 68.1 d | 96.3 c | 15.0 a | -1.6 bc | 14.9 a | 74.6 c | 107.0 b | 9.0 d | -2.6 c | 8.6 d |
| Significance^1^ |  |  |  |  |  | *** | *** | *** | *** | *** | *** | *** | *** | *** | *** |

^1^ *** significant at P≤ 0.001. Different letters within the column indicate significant differences at P=0.05. ^2^ Descriptors (code number is reported within brackets) for *Allium* spp. according to the guidelines of International Plant Genetic Resources Institute (IPGRI) (2001). Bulb shape - 1: Flat; 2: Flat globe; 3: Rhomboid; 4: Broad oval; 5: Globe; 6: Broad elliptic; 7: Ovate; 8: Spindle; 9: High top. Bulb Structure type – 1: Regular multi-fan groups; 2: Regular two-fan groups; 3: Regular multi-cloved radial; 4: Regular quadruple; 5: Regular two-cloved; 6: Irregular. Bulb colour represents the outer skin colour of compound bulb- 1: White; 2: Cream; 3: Beige; 4: White stripes; 5: Light violet; 6: Violet; 7: Dark violet; 99: Other. Clove colour represents the skin colour of the clove: 1: White; 2: Yellow and light brown; 3: Brown; 4: Red; 5: Violet; 99: Other.

^3^ Colour indices based on the CIELAB scale 1976 - L*, lightness/darkness; a* greenish (if negative) and reddish (if positive) tonality; b* bluish (if negative) and yellowish (if positive) tonality; hue angle (h°), the hue; chroma (C*), the vividness/dullness.
