# Supplementary material for: Evaluation of Garlic Landraces from Foggia Province (Puglia Region; Italy)
Source: Foods. 2020 Jun 29;9(7):850. doi: 10.3390/foods9070850 (PMC7404550; doi:10.3390/foods9070850)
Supplement: Supplementary file 1 [file foods-09-00850-s001.zip › Table S2.docx]

**Table S2**. Biometric traits of bulbs and cloves of garlic genotypes.

| Genotype | Acronym | Bulb fresh weight | Bulb equatorial diameter | Cloves per bulb | Clove fresh weight | Clove dry matter |
| --- | --- | --- | --- | --- | --- | --- |
|  |  | (g) | (mm) | (n.) | (g) | (g 100 g^-1^ fw) |
| Spanish white garlic  (Commercial genotype) | ‘CG’ | 39.0 bcd^2^ | 51.1 bcd | 10.7 b | 4.0 ab | 36.4 cd |
| Aglio dei Cortigli  (Landraces) | 'Cortigli' | 56.7 ab | 41.1 d | 11.0 b | 5.2 ab | 38.4 bc |
| Aglio di Peschici  (Landraces) | 'Peschici' | 56.5 ab | 54.6 abc | 19.5 a | 2.9 b | 42.6 b |
| Aglio Rosso di Monteleone di Puglia  (Landraces) | 'Monteleone' | 63.5 a | 60.5 ab | 12.0 b | 5.2 ab | 32.9 d |
| Aglio di Anzano di Puglia  (Landraces) | 'Anzano' | 51.0 ab | 62.9 a | 10.3 b | 4.8 ab | 35.7 cd |
| Aglio Bianco di Panni  (Landraces) | 'Panni' | 30.9 cd | 45.1 cd | 5.0 c | 6.2 a | 36.2 cd |
| Aglio Durevole di Panni  (Landraces) | 'PanniD' | 28.3 d | 14.2 e | 4.7 c | 5.5 ab | 56.5 a |
| Significance^1^ |  | * | *** | *** | * | ** |

^1^ *, **, *** significant at P≤ 0.05, P≤ 0.01, P≤ 0.001, respectively.

^2^ Different letters within the column indicate significant differences at P=0.05.
