# Supplementary figures and images for: Evaluation of Garlic Landraces from Foggia Province (Puglia Region; Italy)
Source: Foods. 2020 Jun 29;9(7):850. doi: 10.3390/foods9070850 (PMC7404550; doi:10.3390/foods9070850)

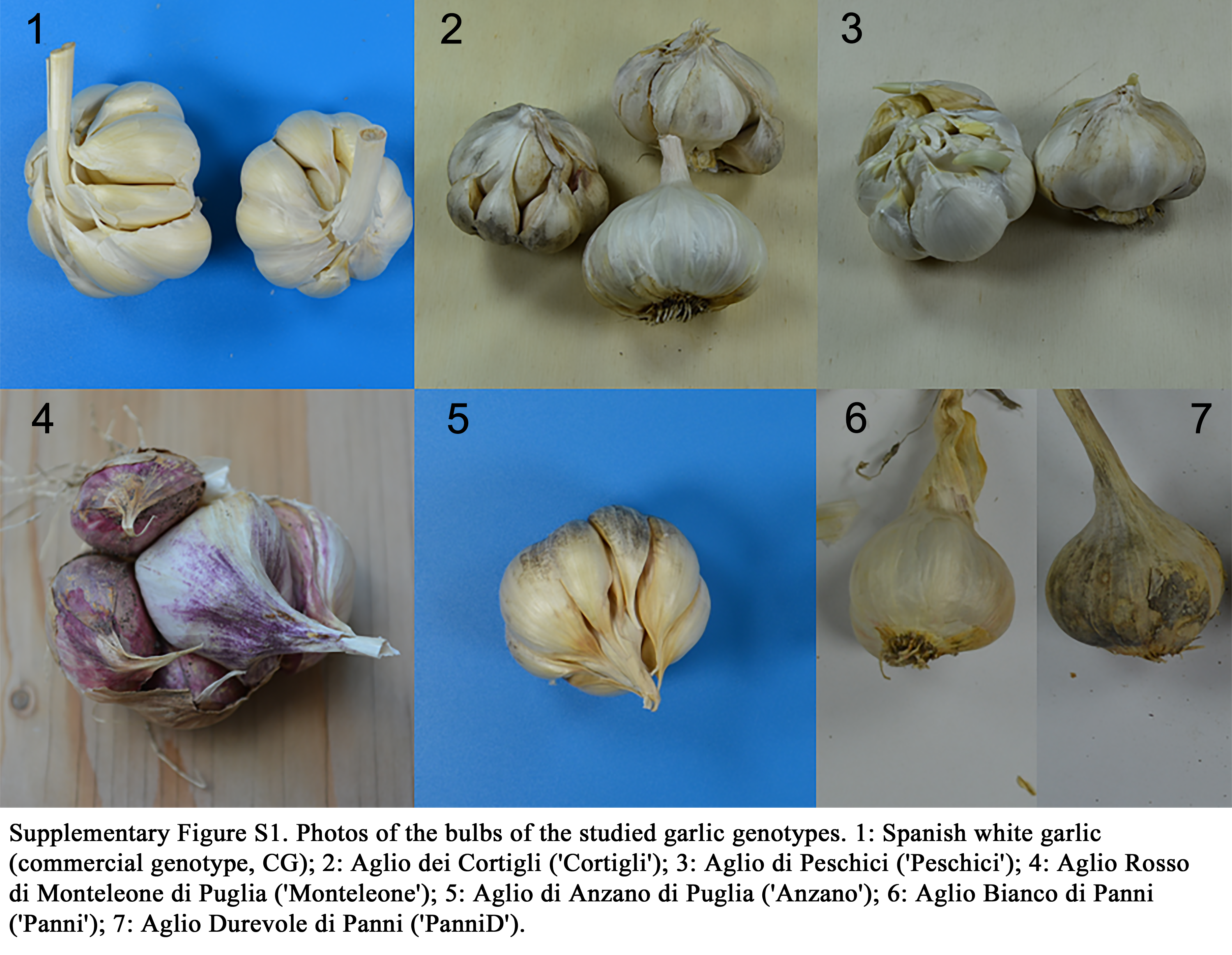

Supplement: Supplementary file 1 [file foods-09-00850-s001.zip › Figure S1.tif]

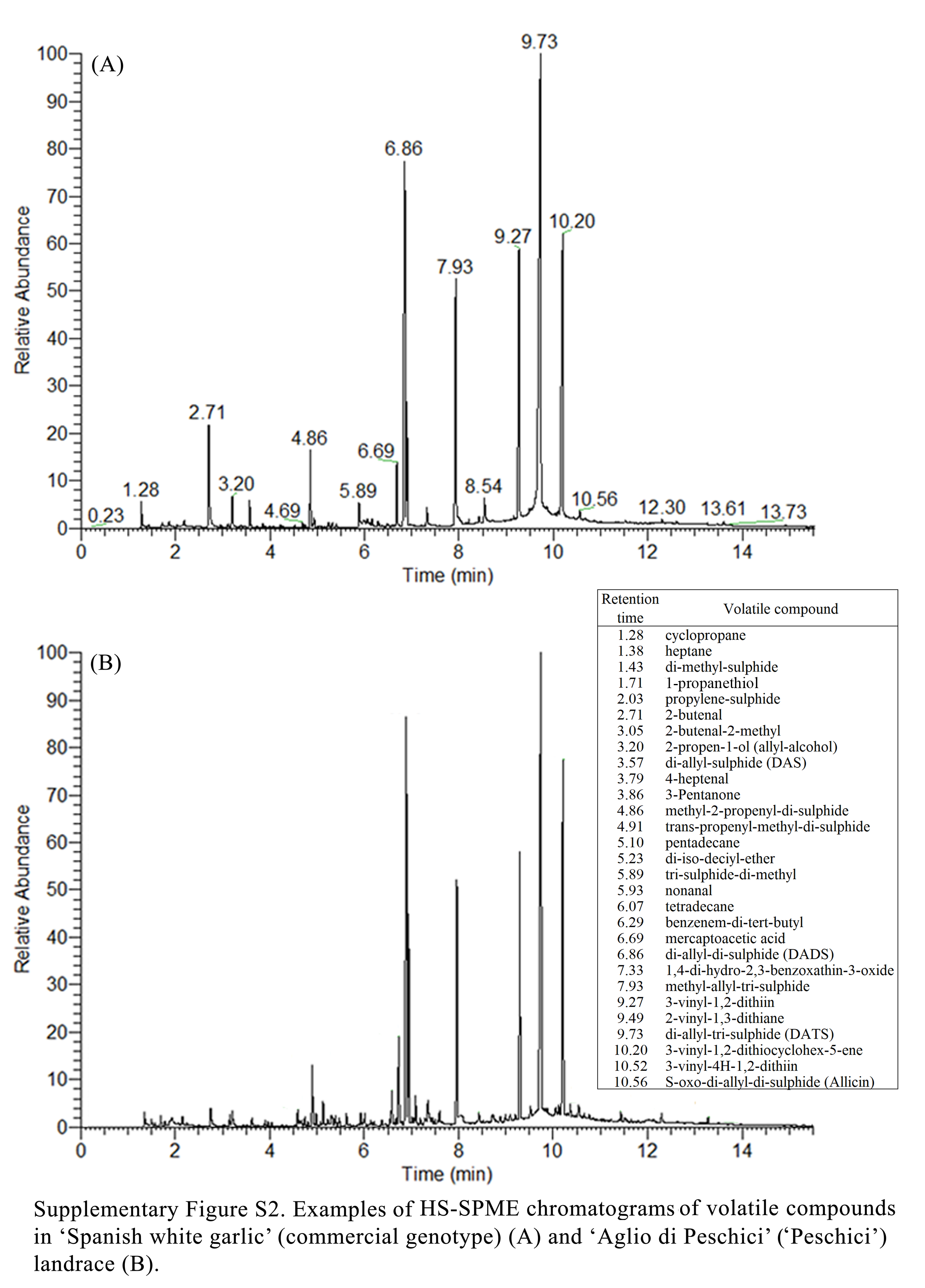

Supplement: Supplementary file 1 [file foods-09-00850-s001.zip › Figure S2.tif]
